# Glutathione peroxidase-1 regulates ASK1-dependent apoptosis via interaction with TRAF2 in RIPK3-negative cancer cells

**DOI:** 10.1038/s12276-021-00642-7

**Published:** 2021-06-22

**Authors:** Sunmi Lee, Eun-Kyung Lee, Dong Hoon Kang, Jiyoung Lee, Soo Hyun Hong, Woojin Jeong, Sang Won Kang

**Affiliations:** 1 Department of Life Science, Ewha Womans University, Seoul, 03760 Korea; 2 The Research Center for Cellular Homeostasis, Ewha Womans University, Seoul, 03760 Korea

**Keywords:** Apoptosis, Cancer prevention

## Abstract

Glutathione peroxidase (GPx) is a selenocysteine-containing peroxidase enzyme that defends mammalian cells against oxidative stress, but the role of GPx signaling is poorly characterized. Here, we show that GPx type 1 (GPx1) plays a key regulatory role in the apoptosis signaling pathway. The absence of GPx1 augmented TNF-α-induced apoptosis in various RIPK3-negative cancer cells by markedly elevating the level of cytosolic H_2_O_2_, which is derived from mitochondria. At the molecular level, the absence of GPx1 led to the strengthened sequential activation of sustained JNK and caspase-8 expression. Two signaling mechanisms are involved in the GPx1-dependent regulation of the apoptosis pathway: (1) GPx1 regulates the level of cytosolic H_2_O_2_ that oxidizes the redox protein thioredoxin 1, blocking ASK1 activation, and (2) GPx1 interacts with TRAF2 and interferes with the formation of the active ASK1 complex. Inducible knockdown of GPx1 expression impaired the tumorigenic growth of MDA-MB-231 cells (>70% reduction, *P* = 0.0034) implanted in mice by promoting apoptosis in vivo. Overall, this study reveals the apoptosis-related signaling function of a GPx family enzyme highly conserved in aerobic organisms.

## Cancer: Antioxidative enzyme protects tumor cells from programmed death

An antioxidative enzyme that plays a critical role in regulating whether cells program their own death offers a promising new target for anti-cancer therapies. Glutathione peroxidase-1 (GPX1) is involved in cleaning up reactive metabolic byproducts such as hydrogen peroxide inside cells. Sang Won Kang and colleagues at Ewha Womans University in Seoul, South Korea, showed that this stress-response enzyme also suppresses the induction of normal programmed cell death mechanisms in a variety of cancer cells. The researchers detailed the molecular partners involved in GPX1-mediated signaling inside cancer cells, and demonstrated that genetically reducing GPX1 expression dramatically reduces tumor growth in a mouse model of breast cancer. Drugs with similar inhibitory effects on GPX1 activity might therefore also help shrink tumors in human cancer patients.

## Introduction

Glutathione peroxidase (GPx) exhibits hydroperoxide-reducing peroxidase activity coupled with glutathione (GSH) and the GSH reductase redox system^1^. Among various isoforms, GPx isoform 1 (GPx1) reduces hydrogen peroxide (H_2_O_2_) and organic hydroperoxides into substrates and is present in the cytosol and mitochondria^2^. GPx has a selenocysteine residue at the active site, and as previously established in vitro kinetics studies, GPx exhibits the peroxidase activity with the millimolar concentration of hydroperoxides, depending on the GSH concentration^3^. Unlike this in vitro activity of GPx, the selenocysteine residue can be converted to dehydroalanine by millimolar concentrations of exogenous H_2_O_2_ in the cellular system^4^. Thus, previous studies have led us to wonder whether GPx1 can play a role in cell death programs, particularly those in which mitochondrial damage produces reactive oxygen species (ROS) exceeding a physiological level^5^.

Apoptosis is a type of programmed cell death that is not only essential for development and organismal homeostasis^6^ but is also detrimental to cancer cells with no or low expression of receptor-interacting protein kinase 3 (RIPK3, also known as RIP3) when they are exposed to death ligands or oxidative stresses^7,8^. In contrast, RIPK3-expressing cancer cells undergo a type of caspase-independent programmed cell death, necroptosis, upon death-ligand stimulation^9^. In necroptosis, RIPK3 interacts with RIPK1 and induces a necrotic cell death through MLKL or mitochondrial energy metabolism^10,11^. Interestingly, RIPK3-dependent necroptosis is countered by caspase-8 and FADD expressions in normal physiology, such as in embryonic development and intestinal epithelial cells, respectively^12–14^.

In RIPK3-negative cancer cells, the apoptosis program is inhibited by elevated NF-κB-dependent survival gene expression^15^. Indeed, the stimulation of cancer cells with tumor necrosis factor (TNF)-α induces strong activation of NF-κB-dependent survival genes^16^. Therefore, blockade or disturbance of the NF-κB pathway results in apoptotic cell death^17^. In an even more compelling outcome, blockade of NF-κB activation is accompanied by sustained JNK activation and robust production of ROS in TNF-α-stimulated cancer cells^18^. Both outcomes are due to the absence of NF-κB target genes, as represented as the JNK pathway-inhibiting molecules GADD45 and XIAP^19,20^, and the antioxidant proteins manganese superoxide dismutase (MnSOD) and ferritin heavy chain^21,22^. In particular, ROS have been thought to be important elements in the intrinsic apoptosis pathway involving mitochondrial outer membrane permeabilization (MOMP)^8^. Since the type of ROS mediating the intracellular apoptosis signal has not been identified, investigating the role of GPx1 in the apoptosis pathway may provide insight into H_2_O_2_ signals that serve as apoptotic messengers.

In this study, we found that GPx1 regulates TNF-α-induced apoptosis in a pathway-selective manner. Unexpectedly, the interaction of GPx1 with TRAF2 was found to regulate the local H_2_O_2_ level, which triggers the activation of apoptosis signal-regulating kinase 1 (ASK1). Thus, our study demonstrates the GPx1-centered signaling module determining cell fate in cancer cells.

## Materials and methods

### Antibodies and reagents

Antibody against Trx1 was purchased from AbFrontier (Seoul, Korea). Antibodies against GPx1 (Cat No. ab22604) and the horseradish peroxidase (HRP)-conjugated Myc epitope (Cat No. ab1326) were purchased from Abcam. Monoclonal antibody against the Myc epitope (9E10 clone, Cat No. 05–419) was purchased from Millipore. Monoclonal antibodies against α-tubulin (Cat No. T5168) and the Flag epitope (M2 clone, Cat No. F1804; HRP-conjugated M2, Cat No. A8592) were purchased from Sigma. Antibodies against cytochrome c (Cat No. 556433) and JNK1 (Cat No. 51–1570GR) were purchased from BD. Antibodies against ERK1 (C-14, Cat No. SC-154 AC), IKKβ (H-4, Cat No. SC-8014), XIAP (H-202, Cat No. SC-11426), and ASK1 (F-9, Cat No. SC-5294) were purchased from Santa Cruz Biotechnology. Anti-ERK1/2-pTpY (Cat No. SC-7383), anti-JNK-pTpY (Cat No. 9255), anti-p38 (Cat No. 9212), anti-p38-pTpY (Cat No. 9211), anti-caspase-3 (Cat No. 9662), and anti-caspase-8 (1C12 clone, Cat No. 9746) antibodies were purchased from Cell Signaling Technology. Antibodies against human cIAP1 (Cat No. AF8181) and cIAP2 (Cat No. AF8171) were obtained from R&D Biosystems. Antibody against human cFLIP (NF6 clone, Cat No. ALX-804–428) was purchased from Alexis. Unless otherwise stated, antibody was diluted for use in western blotting on the basis of the manufacturer’s protocol.

The following small-interfering RNA (siRNA) sequences were used in this study: human GPx1 (#1), GCA AGG UAC UAC UUA UCG A; human GPx1 (#2), UGA AUU CCC UCA AGU ACG U; human GPx1 (#3), GCA ACC AGU UUG GGC AUC A; mouse GPx1, 3′-CGA CAU CGA ACC UGA CAU A-5′; human IKKβ, GGA AGU ACC UGA ACC AGU UUU; and firefly luciferase, CGU ACG CGG AAU ACU UCG A.

DuoLink in situ fluorescence reagents were purchased from Sigma-Aldrich. Caspase-8/Caspase-3 assay kits, JNK inhibitors (SP600125 and JNK inhibitor VIII), p38 inhibitor (SB203580), ERK inhibitor (PD03158), and zVAD-fmk were purchased from Calbiochem (Darmstadt, Germany). Recombinant human TNF-α protein (amino acids 77 to 233) was produced in *E. coli* cells using the expression vector pET17b. The purified soluble TNF-α proteins were determined to be endotoxin-free before use. The pcDNA3.1 plasmid vector encoding human RIPK3 was a kind gift from Dr. Xiadong Wang (NIBS, Beijing, China).

### Cell cultures

HeLa, U2OS, and MDA-MB-231 cells were purchased from American Type Culture Collection (Manassas, VA, USA). The HeLa and MDA-MB-231 cells were cultured in Dulbecco’s modified Eagle’s medium supplemented with 10% fetal bovine serum. The U2OS cells were cultured in McCoy’s 5 A medium supplemented with 10% fetal bovine serum. The cell culture supernatants were periodically tested for mycoplasma contamination using a mycoplasma detection kit (Biotool, USA). HeLa cell lines stably expressing human Bcl-2, human Bcl-X_L_, and a super-repressor IκB lacking an amino-terminal region (amino acids 1–55) have been described elsewhere^23^.

### Immunoblotting and immunoprecipitation assays

The cultured cells were rinsed once with ice-cold phosphate-buffered saline (PBS) and lysed in lysis buffer containing 20 mM HEPES (pH 7.0), 1% Triton X-100, 150 mM NaCl, 10% glycerol, 1 mM EDTA, 2 mM EGTA, 1 mM DTT, 5 mM Na_3_VO_4_, 5 mM NaF, 1 mM AEBSF, aprotinin (5 μg/ml), and leupeptin (5 μg/ml). Tumor tissues were excised from anesthetized mice and homogenized in HEPES-buffered saline containing 10% glycerol, 1 mM EDTA, 2 mM EGTA, 1 mM DTT, 5 mM Na_3_VO_4_, 5 mM NaF, 1 mM AEBSF, aprotinin (5 μg/ml), and leupeptin (5 μg/ml) using a Dounce homogenizer. Tissue homogenates and cell lysates were centrifuged at 15,000 × *g* for 15 min, and protein concentrations were determined by Bradford assay (Pierce). Protein samples were mixed with SDS sample buffer and boiled for 5 min. The proteins were separated by SDS-PAGE and transferred onto nitrocellulose membranes by electroblotting for 1 h. The membranes were blocked with 5% bovine serum albumin (BSA) or 5% dry skimmed milk in Tris-buffered saline containing 0.05% (v/v) Tween-20 (TBST) for 2 h and incubated with the appropriate primary antibody in blocking buffer for 2 h at room temperature. After washing three times with TBST, the membranes were incubated with HRP-conjugated secondary antibody (Amersham Biosciences) in blocking buffer. The immunoreactive bands were detected with an enhanced chemiluminescence kit (AbFrontier, Korea) and quantified by a LAS-3000 imaging system (Fuji Film, Japan). When necessary, the membranes were stripped by shaking them for 60 min at 37 °C in 67 mM Tris (pH 6.7), 2% SDS, and 100 mM β-mercaptoethanol and reprobed with an appropriate pan-antibody.

For immunoprecipitation assays, the clarified cell lysates (0.5–1 mg protein) were precleared with 10 μl of protein-A/G Sepharose 4 Fast Flow beads (Amersham Biosciences) for 1 h. The supernatant was incubated overnight with 3 μg of the appropriate antibody with rotation and precipitated by the addition of 30 μl of protein-A/G beads at 4 °C and mixing for an additional 3 h. The beads were washed three times with 1 ml of lysis buffer and subjected to immunoblotting.

### Plasmid construction and site-directed mutagenesis

Retroviral vectors (pQ-CXIX) expressing wild-type (WT) Myc-tagged mouse GPx1 were prepared by PCR cloning. The PCR product encoding GPx1 includes the part of the 3’-UTR containing the SECIS sequence, which is necessary for selenocysteine incorporation. Site-directed mutagenesis for amino acid substitution was performed using a QuikChange kit (Stratagene). The double-stranded primer for the Sec47S mutant of mouse GPx1 was (sense) 5′-GTCGCGTCTCTCTCAGGCACCACGATCCG-3′; the mutated nucleotide is underlined. The pcDNA3.1 vectors encoding wild-type human TRAF2 and truncated mutants were kind gifts from Dr. Soo-Young Lee (Ewha Womans University, Seoul, Korea). All constructs and mutations were verified by nucleotide sequencing.

### Apoptosis assays

Unless otherwise stated, the cancer cells were stimulated with TNF-α (10 ng/ml) plus cycloheximide (10 μg/ml) for 6 h. The stimulated cells were washed once in PBS and incubated at 37 °C for 2 min in 0.05% trypsin-EDTA. cells were gently removed by pipetting and added to 5-ml FACS tubes containing the culture medium and PBS wash. The cells were then centrifuged for 3 min, washed with cold PBS, and the final cell pellets were stained using an annexin V-FITC apoptosis detection kit I (BD Pharmingen) according to the manufacturer’s protocol. Briefly, cells were incubated with annexin V-FITC for 20 min followed by propidium iodide (PI) for 5 min on ice. The stained cells were analyzed using a FACSCalibur system (Becton Dickinson). The percentage of apoptotic cells was determined with the equation [100 - percent of PI-negative/annexin-V-negative cells].

### In vitro ASK1 activity assay

HeLa cells were stimulated with TNF-α (20 ng/ml) plus cycloheximide (10 μg/ml) for 2 h, rinsed once with ice-cold PBS, and lysed in lysis buffer. The cell lysates were precleared with 10 μl of protein A/G agarose beads for 1 h. The cleared lysates were incubated with 4 μg of ASK1 antibody for 2 h and then further incubated with 30 μl of protein-A/G beads at 4 °C overnight with rotation. The agarose beads were washed three times with 1 ml of lysis buffer and then washed twice with reaction buffer (20 mM HEPES, pH 7.4; 5 mM MgCl_2_; 10 mM β-glycerophosphate; 0.1 mM Na_3_VO_4_; 2 mM NaF, and 1 mM dithiothreitol). The bead suspension was incubated in 30 μl of reaction buffer containing 10 μM ATP, 0.6 μCi [γ-^32^P] ATP, and 1 μg of myelin basic proteins at 30 °C for 30 min. The reaction was quenched by adding 10 μl of 5 × SDS sample buffer. The radiolabeled proteins were resolved on a 12% denaturing gel and detected by autoradiography.

### Measurement of intracellular ROS levels

Intracellular ROS generation was assessed using 5,6-chloromethyl-2′,7′-dichlorodihydrofluorescein diacetate (CM-H_2_DCFDA) (Molecular Probes). Cells (3 × 10^5^) were plated on 35-mm dishes and cultured for 24 h. The cells were deprived of serum for 6 h and stimulated by incubation with TNF-α (20 ng/ml) plus cycloheximide (10 μg/ml) for multiple durations in phenol red-free media. After stimulation, the cells were rinsed once with 2 ml of KREB’s Ringer solution and incubated for 5 min with 5 μM CM-H_2_DCFDA. The plate was mounted, and DCF fluorescence images were immediately acquired by fluorescence microscopy (Axiovert200, Zeiss). The fluorescence intensities were measured using a Scion imaging system.

Intracellular H_2_O_2_ generation was also assessed using a circularly permuted yellow fluorescent protein, called HyPer-Cyto, inserted into the regulatory domain of OxyR. HeLa cells were transfected with the plasmid vector encoding HyPer-Cyto (Evrogen Co., Russia) and incubated for 12 h. The transfected cells were rinsed with DMEM without phenol red and then mounted for a confocal laser scanning microscopy (with an LSM 880 microscope with Airyscan, Zeiss). Fluorescence images were acquired at 510 nm with excitation at 405 and 488 nm. The ratio of the fluorescence intensities obtained at 405- and 488-nm excitation were calculated.

The lipid hydroperoxide (LOOH) level was measured with a C11-BODIPY^581/591^ ratiometric fluorescent probe. Cells were transfected with the indicated siRNAs, incubated for 36 h, rinsed with ice-cold PBS, and fixed with 3.7% formaldehyde. The fixed cells were permeabilized with 0.1% Triton X-100 in PBS for 5 min and stained with 1 μM C11-BODIPY for 30 min at 37 °C The green (oxidized) and red (reduced) fluorescence images were simultaneously acquired using a Zeiss LSM 880 confocal microscope.

### Proximity ligation assay

Cells were grown on glass cover slips for 36 h, fixed with 3.7% paraformaldehyde for 10 min, and permeabilized with 0.1% Triton X-100 in PBS for 5 min. The fixed cells were incubated with a drop of blocking solution for 30 min at 37 °C. Anti-TRAF2 and anti-GPx1 antibodies were diluted 1:1000 (for PLUS and MINUS, respectively) in block buffer containing 1 × Duolink assay reagent. Samples were incubated overnight with antibody solutions at 4 °C. The polymerase chain reaction was performed according to the manufacturer’s instructions. After polymerase chain reaction, nuclei were stained with DAPI for 10 min at room temperature. Images were taken using a Zeiss LSM 880 confocal microscope.

### Construction of an inducible MDA-MB-231 cell line

MDA-MB-231 cells were transfected with a lentiviral pTRIPZ vector encoding GPx1-specific shRNA^mir^ under a Tet-on inducible promoter (V3THS_413713; GE healthcare). The transfected cells were subcultured for two weeks in a complete medium containing 1.5 μg/ml puromycin. The selected cells were trypsinized, diluted, and replated onto a 96-well culture plate at the single-cell level. Each cell was cultured until a visible clone was observed and then expanded on six-well culture plates. The individual clones were tested for GPx1 knockdown after treatment with doxycycline (5 μg/ml) for 24 h. The knockdown level of GPx1 protein was evaluated by immunoblot analysis.

### Tumor xenograft study

All mouse experiments were approved by the Institutional Animal Care and Use Committee (IACUC) of Ewha Womans University, South Korea, and conformed to ARRIVE guidelines. MDA-MB-231 cells stably harboring inducible GPx1 shRNA were treated with 5 μg/ml doxycycline for 24 h, trypsinized, and then resuspended in 200 μl of PBS. The suspended cells (2.5 × 10^6^ cells per mouse) were subcutaneously injected into the back side of Balb/c *nu/nu* mice (4-week-old males) anesthetized by inhalation of isoflurane gas (N_2_O:O_2_/70%:30%). Doxycycline was intraperitoneally injected once a week starting one week before the injection of cancer cells. To induce tumor cell apoptosis, recombinant mouse TNF-α (40 μg/kg) was intravenously injected twice weekly from 7 days to 38 days. Tumor volume was measured once every three days with a caliper and calculated according to the formula *V* = *a* *×* *b*^2^/2, where *a* and *b* denote long and short superficial diameters, respectively. Tumor tissues were removed and weighed after the mice were sacrificed 40 days postinjection of the cells.

### Statistical analysis

Data analysis was performed with Student’s *t*-tests for comparisons between two groups or two-way ANOVAs with Tukey’s ‘honestly significant difference’ post hoc test for multiple groups (SPSS 12.0 K for Windows, SPSS, Chicago, IL, USA) to determine the statistical significance (*P* value). The data are presented as the means ± standard deviation (SD). *P* < 0.05 was considered statistically significant.

## Results

### GPx1 negatively regulates TNF-α-induced apoptosis

**Fig. 1:**
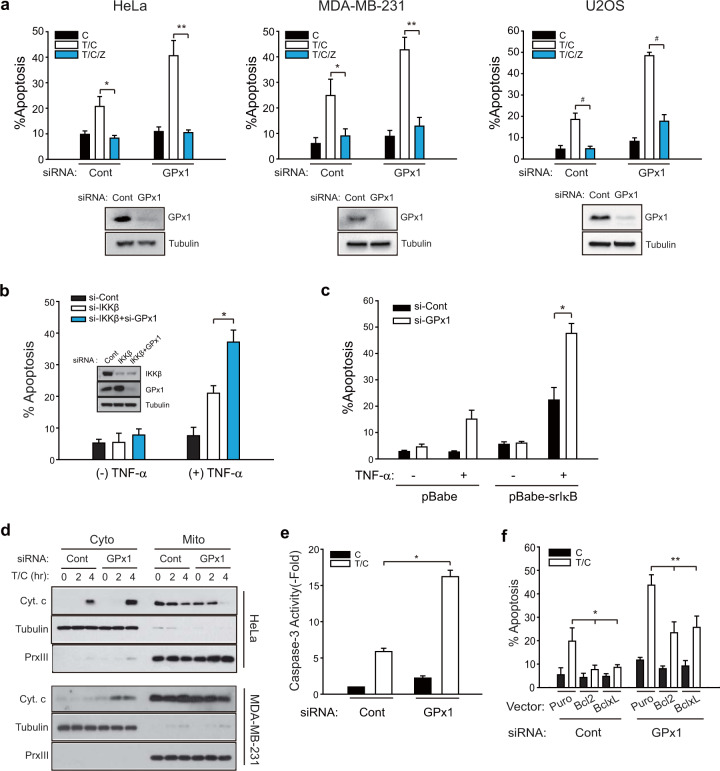
GPx1 suppresses the TNF-α-induced apoptosis of RIPK3-negative cancer cells. **a** HeLa, MDA-MB-231, and U2OS cells were transfected with a mixture of three siRNAs specific to GPx1. After 6 h of treatment with cycloheximide (C) alone or TNF-α plus cycloheximide (T/C), the cells were labeled with propidium iodide (PI) and annexin-V and subjected to FACS analysis. The pan-caspase inhibitor z-VAD-fmk (Z) was added as a pretreatment for 1 h before T/C stimulation. The data in the graph are the means ± SD of the percentage of apoptotic cells (*n* = 3, ^**^P < 0.005, ^**^P < 0.001, and ^#^*P* < 0.0001). Firefly luciferase-specific siRNA was used as a control (Cont). **b**, **c** IKKβ-depleted (**b**) or super-repressor IκBα (srIκB)-expressing HeLa cells (**c**) were transfected with siRNA (#3) against GPx1 and treated with TNF-α (10 ng/ml) for 8 h. The data in the graph are the means ± of the percent of apoptotic cells (*n* = 3, *P < 0.001). The knockdown levels of the GPx1 and IKKβ proteins were verified by immunoblotting. **d** HeLa and MDA-MB-231 cells were transfected with GPx1 siRNA (#3) for 36 h and subjected to subcellular fractionation following T/C stimulation. Cytosolic (Cyto) and mitochondrial (Mito) fractions were subjected to immunoblot analysis for determining cytochrome c (Cyt.c) release. A representative blot is shown (*n* = 3). Tubulin and peroxiredoxin III (PrxIII) are cytosolic and mitochondrial markers, respectively. **e** siRNA-transfected HeLa cells were stimulated with T/C and lysed for use in a caspase-3 activity assay. The data in the graph are the means ± SD of the fold change of caspase-3 activity (*n* = 3, ^*^P < 0.0001). **f** Vector control (Puro)-, Bcl-2-, and Bcl-xL-expressing HeLa cell lines were transfected with either control or GPx1 siRNA, stimulated with T/C for 8 h, and subjected to apoptosis assays. The data in the graph are the means ± SD of the percent of apoptotic cells (*n* = 3, ^*^P < 0.001 and ^**^P < 0.0001).

Since GPx1 is broadly expressed in human tumor tissues^24^, we examined the effect of GPx1 depletion in cancer cells undergoing TNF-α-induced apoptosis. To specify the canonical apoptosis pathway, receptor-interacting kinase 3 (RIPK3)-negative cancer cell lines, such as HeLa, MDA-MB-231, and U2OS cells, were selected (Supplementary Fig. S1a) and stimulated with TNF-α in the presence of cycloheximide, which inhibits the synthesis of NF-κB target proteins^25^. When these cancer cells were transfected with a siRNA specific to GPx1, the resulting depletion of GPx1 caused markedly augmented TNF-α-induced apoptosis of all the cancer cells selected, which were then completely blocked by treatment with a pan-caspase inhibitor (zVAD-fmk) (Fig. 1a). Notably, FACS and TUNEL analyses indicated that GPx1 depletion increased the apoptotic fractions of dead cells (Supplementary Fig. S1b, c). However, GPx1 depletion did not induce TNF-α-induced apoptosis in MCF10A normal mammary epithelial cells (Supplementary Fig. S1d). Because MCF10A is a RIPK3-negative normal cell type^26^, we additionally tested TNF-α-induced apoptosis of L929 mouse fibroblasts expressing RIPK3 and confirmed that GPx1 had no effect on the apoptosis rate of these noncancerous cells regardless of RIPK3 expression. To confirm the on-target effect of the siRNA used, we tested three independent siRNA sequences specific to GPx1. Transfection with three different siRNAs targeting GPx1 similarly increased the rate of TNF-α-induced apoptosis of HeLa cells (Supplementary Fig. S1e), which indicates that GPx1 is indeed a negative regulator of apoptosis. Since cycloheximide treatment is an artificial means of blocking NF-κB gene expression, we verified the antiapoptotic function of GPx1 in HeLa cell lines either depleted of IκB kinase β (IKKβ) or overexpressing a super-repressor form of IκB lacking the S32/S36-harboring N-terminal region (srIκB)^27^. As expected because of previous results obtained in the apoptosis field^28,29^, TNF-α stimulation alone induced apoptosis of the HeLa cell with abrogated NF-κB activation, which was constantly augmented by the GPx1 depletion (Fig. 1b, c). The results confirmed that GPx1 depletion accelerates the canonical apoptosis pathway induced by blocking NF-κB-dependent gene expression. In addition, we showed that GPx1 depletion had no effect on the necroptosis induced by cotreatment of TNF-α and zVAD in the cells with stable expression of RIPK3, such as Jurkat human T cells and L929 mouse fibroblast cells, or in the HeLa cells transiently expressing human RIPK3 (Supplementary Fig. S1f, g), which strongly supported the supposition that GPx1 has a specific function in apoptosis, not necroptosis.

Next, we dissected the molecular pathway by which GPx1 regulates TNF-α-induced apoptosis. First, GPx1 depletion augmented the mitochondrial release of cytochrome c and subsequent activation of caspase-3 in TNF-α-stimulated HeLa and MDA-MB-231 cells compared to control cells (Fig. 1d, e), indicating GPx1-dependent regulation of the intrinsic apoptosis pathway. Second, the involvement of mitochondria was tested by determining Bcl-2 and Bcl-xL expression (Fig. 1f). The apoptosis assay showed that Bcl-2 and Bcl-xL expression completely inhibited apoptosis of control HeLa cells, which indicates that TNF-α induced mitochondria-dependent (intrinsic) apoptosis. However, in GPx1-depleted cells, the expression of the Bcl-2 proteins reduced the level of TNF-α-induced apoptosis by ~50%, which means that GPx1 depletion also enhanced mitochondria-independent apoptosis in addition to the intrinsic pathway. Notably, GPx1 depletion itself did not change the level of Bcl-2 protein expression. Third, GPx1 depletion also enhanced the apoptosis induced by TNF-α plus a Smac mimetic (BV6), which further supports the involvement of GPx1 in the mitochondria-independent pathway (Supplementary Fig. S2). These data suggest that GPx1 regulates apoptosis signaling upstream of mitochondria.

### GPx1 is a bona fide thiol peroxidase that regulates cytosolic H_2_O_2_ level

**Fig. 2:**
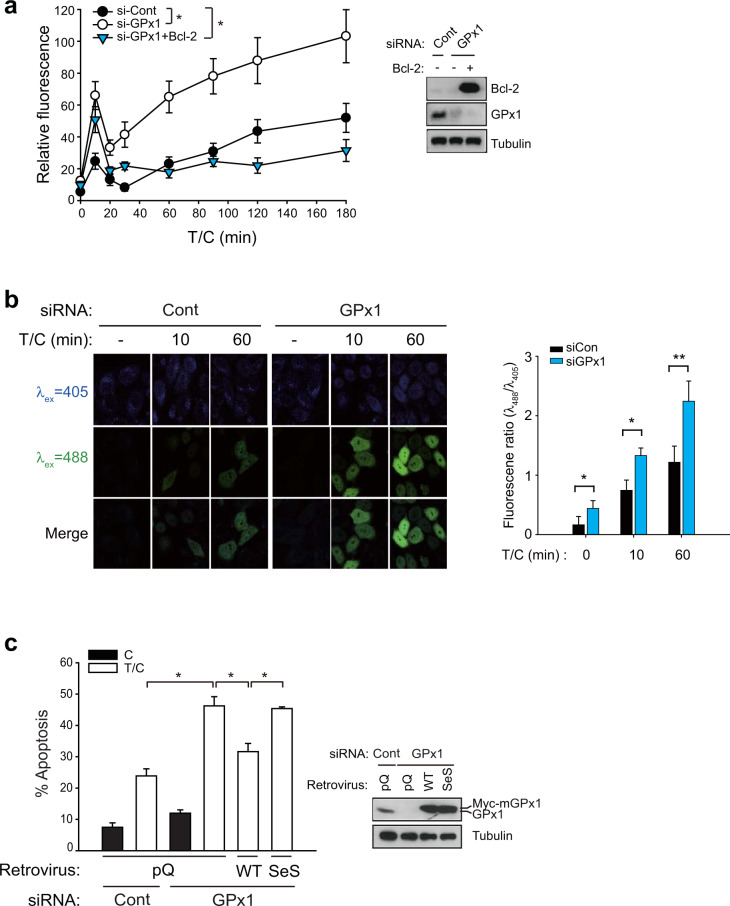
GPx1 is critical for regulating the intracellular H_2_O_2_ level. **a** Intracellular ROS levels were determined using 2′,7′-dichlorodihydrofluorescein diacetate (DCFH-DA) treatment of siRNA-transfected HeLa cells following T/C stimulation. Bcl-2-overexpressing HeLa cells were analyzed for comparison. The data in the graph are the means ± SD of relative DCF fluorescence intensities from 80 to 100 cells (*n* = 3, ^*^P < 0.0001 with repeated-measures ANOVA). **b** Intracellular H_2_O_2_ levels were measured in HyPer-expressing HeLa cells. Fluorescence images were taken of the HyPer-expressing HeLa cells following T/C stimulation. The data in the graph are the means ± SD of the ratio of fluorescence intensities at 488 and 405 nm (*n* = 15–25 cells). Representative images are shown. **c** siRNA-transfected HeLa cells were infected with the indicated retroviruses encoding WT mouse GPx1 or an inactive mutant (SeS). The apoptosis rate was measured after T/C stimulation for 6 h. The data in the graph are the means ± SD of the percent of apoptotic cells (*n* = 3, ^**^P < 0.005). Immunoblots show the level of knockdown and rescued expression of GPx1. Empty retroviral vector (pQ) was used as a control.

Since GPx1 is the cytosolic thiol peroxidase that eliminates H_2_O_2_, we examined the change in the cytosolic ROS level in HeLa cells undergoing TNF-α-induced apoptosis using a chemical probe: carboxymethyl-2′,7′-dichlorofluorescin (DCF) diacetate^30^. Fluorescence microscopic analyses demonstrated that the cytosolic ROS level was increased in the HeLa cells by TNF-α stimulation, which was dramatically enhanced by GPx1 depletion (Fig. 2a). Consistent with our previous study^23^, ROS generation was biphasic: a small transient peak was followed by a persistent and robust second peak. Again, Bcl-2 expression reduced only the second peak representing ROS enhancement by GPx1 depletion, which confirms that the second peak can be attributed to mitochondrial pathways. To determine whether H_2_O_2_ is the ROS regulated by GPx1, we performed a microscopic analysis of HeLa cells expressing an H_2_O_2_-specific fluorescence protein named HyPer-Cyto^31^. The results showed that TNF-α stimulation increased the green fluorescence at an excitation wavelength of 488 nm, indicating the oxidation of HyPer by H_2_O_2_ (Fig. 2b). Alkaline-induced HyPer fluorescence can be excluded as a contributor to the fluorescence observed because intrinsic apoptosis involves cytosolic acidification^32,33^. Consistent with the DCF fluorescence level, both early and late HyPer fluorescence levels after 10 min and 60 min of stimulation were significantly enhanced by GPx1 depletion, which indicated that H_2_O_2_ was elevated in the GPx1-depleted cells. In addition, we tested lipid peroxidation in the TNF-α-stimulated cells using a C11-BODIPY probe. Neither TNF-α stimulation nor GPx1 depletion induced lipid peroxidation (Supplementary Fig. S3). In contrast, a parallel control experiment showed that the ferroptosis inducer erastin strongly induced lipid peroxidation^34^. Overall, these results imply that GPx1 is the major peroxidase controlling the cellular H_2_O_2_ level in apoptotic cells.

To further document that GPx1 activity is involved in anti-apoptosis effects, we performed rescue experiments using a retroviral expression system of mouse GPx1 resistant to human siRNA knockdown. Wild-type (WT) and an activity-dead mutant (SeS), in which selenocysteine is used to replace the active-site serine residue, were expressed in HeLa cells depleted of endogenous GPx1. The apoptosis assay showed that the rescued expression of WT enzymes, not inactive mutant, diminished apoptosis that had been increased upon GPx1 depletion (Fig. 2c). Thus, the data indicate that the H_2_O_2_-eliminating activity of GPx1 is essential for controlling apoptosis. In addition, the rescue experiment verified the specific on-target effect of the siRNA used in this study.

### GPx1 targets sustained JNK activation in TNF-α-induced apoptotic pathways

**Fig. 3:**
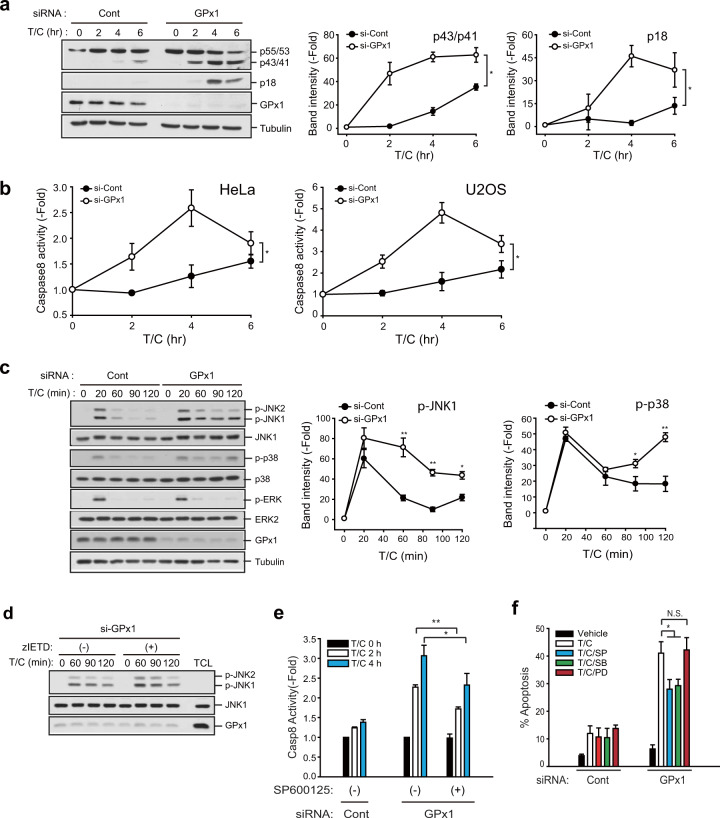
GPx1 suppresses activation of both JNK and caspase-8. **a** siRNA-transfected HeLa cells were stimulated with T/C and lysed for use in immunoblotting. The immunoblot bands for cleaved caspase-8 (with intermediary p43/41 and active p18 fractions) were quantified and normalized to the intensity of α-tubulin bands. The data in the graph are the means ± SD of the fold change of band intensity (*n* = 3, ^*^P < 0.0001 with repeated-measures ANOVA). Immunoblots with α-tubulin used as the loading control. Full-length procaspase-8 is indicated by its size (p55/53). **b** siRNA-transfected HeLa cells were stimulated with T/C and lysed for use in caspase-8 activity assay. The data in the graph are the means ± SD of the fold change of caspase-8 activity (*n* = 3, ^*^P < 0.0001 with repeated-measures ANOVA). **c** siRNA-transfected HeLa cells were stimulated with T/C and lysed for use in immunoblotting. The immunoblot bands of the phosphorylated MAPKs were quantified and normalized to the intensity of the corresponding MAPK bands. The data in the graph are the means ± SD of the fold change of band intensity (*n* = 3, ^*^P < 0.05, ^**^P < 0.001 versus the matched control). Immunoblots with α-tubulin used as the loading control. **d** siRNA-transfected HeLa cells were pretreated with vehicle control or caspase-8 inhibitor z-IETD-fmk for 30 min, followed by T/C stimulation, and lysed for use in immunoblotting. A representative blot is shown (*n* = 3). Total cell lysates (TCLs) were loaded to determine the level of endogenous proteins. Dimethyl sulfoxide (DMSO) was used as the vehicle control. **e** siRNA-transfected HeLa cells were pretreated with vehicle control or the JNK inhibitor SP600125 for 30 min, stimulated with T/C, and lysed for use in caspase-8 activity assays. The data in the graph are the means ± SD of the fold change of caspase-8 activity (*n* = 3, ^*^P < 0.05, ^**^P < 0.0005). **f** siRNA-transfected HeLa cells were pretreated with vehicle control or MAPK inhibitors (10 μM each) for 30 min. The apoptosis rate was measured after T/C stimulation for 6 h. The data in the graph are the means ± SD of the percent of apoptotic cells (*n* = 3, ^*^P < 0.0001). SP600125 (SP), SB203880 (SB), and PD03158 (PD) are specific inhibitors of JNK, p38, and ERK, respectively. NS not significant.

To determine an H_2_O_2_-sensitive signaling pathway, we investigated the activation of MAP kinases or the initiator caspase-8, which coordinate both intrinsic and extrinsic apoptosis pathways^16^. Upon TNF-α stimulation, procaspase-8 (p55/53) is recruited to the FADD-containing death complex and then activated by cleavage into intermediary (p43/41) and mature (p18) forms^35,36^. A western blot analysis showed that depletion of GPx1 drastically accelerated caspase-8 cleavage and activation in TNF-α-stimulated HeLa cells (Fig. 3a). Moreover, a caspase assay using a fluorogenic substrate showed that the kinetics of the caspase-8 activity was increased similar to that of caspase-8 cleaved in GPx1-depleted HeLa and U2OS cells, compared to that of control cells (Fig. 3b). It should be noted that caspase-8 activity was substantially decreased at later time points, as indicated by the intensity of the p18 fragment of active caspase-8. Subsequently, we examined TNF-α-induced MAPK activation. The results showed that depletion of GPx1 enhanced sustained JNK and p38 activation, but not ERK activation, in TNF-α-stimulated HeLa cells compared to control cells (Fig. 3c). In MDA-MB-231 breast cancer cells, depletion of GPx1 enhanced only sustained JNK activation (Supplementary Fig. S4a). We further determined the hierarchical relationship between JNK and caspase-8 by performing crosscheck assays with specific inhibitors. The results showed that the caspase-8 inhibitor z-IETD-fmk did not affect sustained JNK activation in GPx1-depleted HeLa cells, whereas the JNK inhibitor (SP600125) significantly blocked caspase-8 activation in GPx1-depleted HeLa cells (Fig. 3d, e). Moreover, JNK inhibition consistently reduced the apoptosis rate that had been enhanced by GPx1 depletion in both HeLa and MDA-MB-231 cells (Fig. 3f and Supplementary Fig. S4b). Since the role played by JNK was vital to these results, we confirmed the reduction of GPx1-dependent apoptosis by treating cells with another specific JNK inhibitor, JNK inhibitor VIII, which is an aminopyridine compound (Supplementary Fig. S4c). Collectively, the results indicate that GPx1-mediated apoptosis regulation targets sustained JNK activation upstream of caspase-8.

### GPx1 regulates apoptosis via the ASK1-JNK-cFLIP-Caspase-8 pathway

**Fig. 4:**
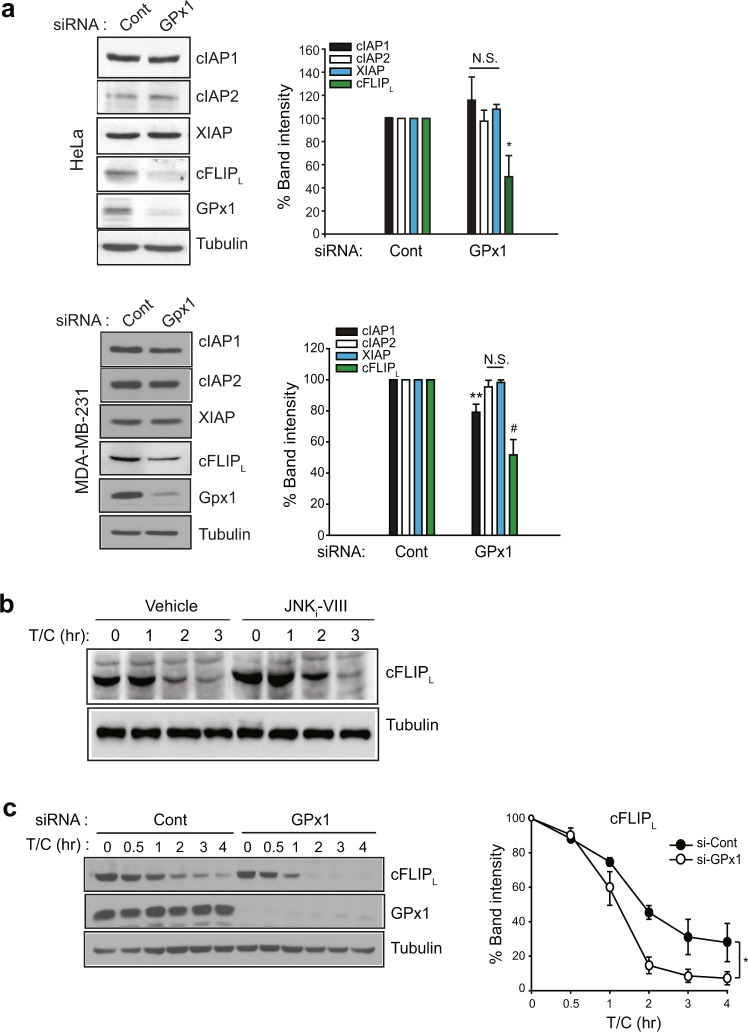
GPx1 absence selectively reduces the cFLIP level. **a** HeLa and MDA-MB-231 cells were transfected with the indicated siRNAs for 24 h and lysed for use in immunoblotting. The immunoblot bands were quantified and normalized to the intensity of α-tubulin bands. The data in the graph are the means ± SD of percent of the band intensities representing GPx1 siRNA-transfected cells relative to those representing control siRNA-transfected cells (*n* = 3, ^*^P < 0.005, ^**^P < 0.002, and ^#^*P* < 0.001 versus the corresponding control samples). Immunoblots with α-tubulin used as the loading control. NS not significant. **b** HeLa cells were pretreated with the JNK inhibitor VIII (JNKi-VIII, 10 μM) for 30 min and stimulated with T/C for the indicated times. The cells were lysed for use in immunoblotting. A representative blot is shown (*n* = 3). **c** siRNA-transfected HeLa cells were stimulated with T/C and lysed at the indicated times for use in immunoblotting. The immunoblot bands for cFLIP_L_ were quantified and normalized to the intensity of α-tubulin bands. The data in the graph are the means ± SD of percent of cFLIP_L_ intensity in T/C-stimulated cells relative to that in unstimulated cells (*n* = 3, ^*^P < 0.0001 with repeated-measures ANOVA). An immunoblot with α-tubulin used as the loading control.

**Fig. 5:**
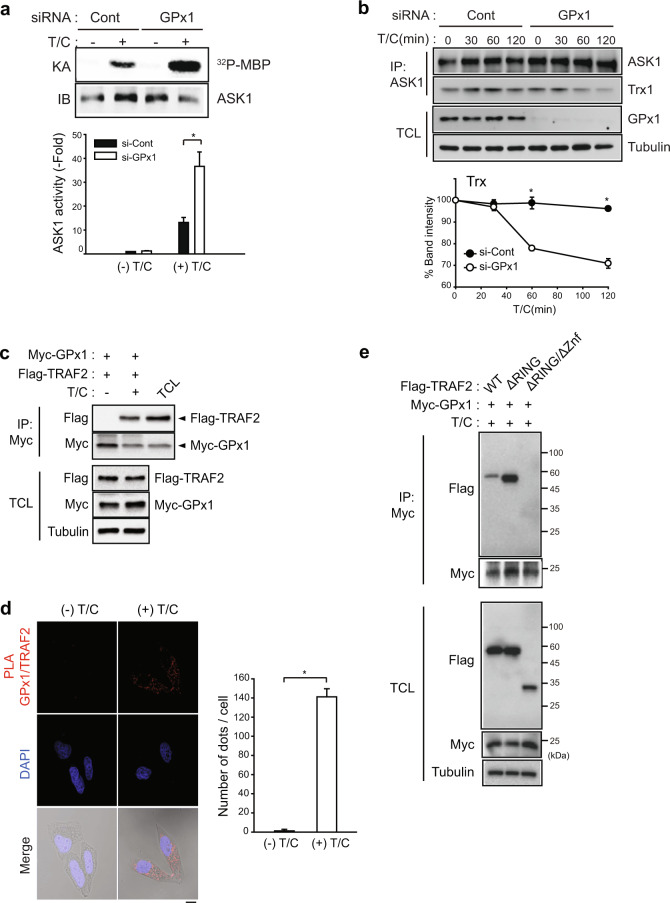
GPx1 regulates ASK1 activation via interaction with TRAF2. **a** siRNA-transfected HeLa cells were stimulated with T/C for 2 h and lysed for use in an immunoprecipitation (IP) assay with an anti-ASK1 antibody. Immunoprecipitates were subjected to an in vitro kinase assay (KA). The data in the graph are the means ± SD of the ^32^P-radioactive band intensities in the autoradiograph normalized to the ASK1 level in the immunoblot (*n* = 3, ^*^P < 0.003). **b** siRNA-transfected HeLa cells were stimulated with T/C for the indicated times and lysed for use in an immunoprecipitation assay with an anti-ASK1 antibody. The data in the graph are the means ± SD of the relative intensities of the Trx bands normalized to that of the ASK1 band (*n* = 3, ^*^P < 0.0002). Total cell lysate (TCL) was loaded to determine the levels of endogenous proteins. (**c**) HeLa cells were cotransfected with expression vectors for Myc-tagged GPx1 and Flag-tagged TRAF2, stimulated with T/C for 2 h, and lysed for use in an immunoprecipitation assay with an anti-Myc antibody. Total cell lysate (TCL) was loaded to determine the levels of endogenous proteins. A representative blot is shown (*n* = 3). **d** HeLa cells were stimulated with T/C for 1 h and then subjected to an in situ proximity ligation assay (PLA) with anti-GPx1 and anti-TRAF2 antibodies. Cell images (Duolink) were taken by confocal microscopy. The data in the graph are the means ± SD of the average number of red fluorescent dots per cell as determined with 40–60 cells (*n* = 3, ^*^P < 0.00001). Scale bar, 10 μm. **e** HeLa cells were cotransfected with expression vectors for Myc-tagged GPx1 and Flag-tagged TRAF2 mutants, stimulated with T/C for 2 h, and lysed for use in an immunoprecipitation assay with an anti-Myc antibody. Total cell lysate (TCL) was loaded to measure the levels of endogenous proteins. A representative blot is shown (*n* = 3). Znf, zinc finger.

Since sustained JNK activation leads to cFLIP_L_ degradation via the E3 ligase Itch^37^, we examined the level of the endogenous caspase-8 inhibitor cFLIP_L_, which is a downstream effector of JNK. Among various caspase-inhibiting proteins, GPx1 depletion selectively reduced the level of cFLIP_L_, but not other IAPs, in HeLa and MDA-MB-231 cells (Fig. 4a). A slight reduction in cIAP1 observed in MDA-MB-231 cells was presumed to be a result of a cell type-dependent effect. In addition, we confirmed that TNF-α-induced cFLIP_L_ degradation was downstream of JNK (Fig. 4b). Subsequently, TNF-α-induced cFLIP_L_ degradation was kinetically much faster in GPx1-depleted HeLa cells than in control cells (Fig. 4c). Next, we examined the activity of apoptosis-signal kinase 1 (ASK1), a key upstream kinase for sustained JNK activation^38^, because its activation is known to be controlled by Trx1, which senses H_2_O_2_^39^. The in vitro kinase assay showed that TNF-α-induced ASK1 activation was strongly enhanced in GPx1-depleted HeLa cells compared to control cells (Fig. 5a). Furthermore, GPx1 depletion accelerated Trx dissociation from the ASK1 complex (Fig. 5b), which supports the enhanced TNF-α-induced ASK1 activation. To understand the molecular mechanism underlying the GPx1 effect on ASK1 activation, we performed a protein interaction study but failed to observe any direct interaction between GPx1 and ASK1. Since ASK1 activation requires oligomerization with TRAF2^40^, we addressed the involvement of TRAF2. An immunoprecipitation experiment showed that TNF-α stimulation induced the protein-protein interaction of GPx1 and TRAF2 in HeLa cells (Fig. 5c). To confirm GPx1 and TRAF2 binding in situ, we performed a proximity ligation assay (PLA), which enabled us to measure the protein-protein interaction quantitatively at the single-cell level^41^. The PLA images showed that GPx1 was associated with TRAF2 upon TNF-α stimulation in the cytoplasm (Fig. 5d). The domain-mapping study further demonstrated that GPx1 was bound to the Zn finger domain of TRAF2 (Fig. 5e). It was noted that the lack of an N-terminal RING domain upstream of the Zn finger domain promoted GPx1 binding to TRAF2. Collectively, we concluded that GPx1 functions as the master regulator of the ASK1-JNK-cFLIP-caspase-8 axis in apoptosis pathways via stimulation-dependent interaction with TRAF2 in cancer cells.

### GPx1 is essential for cancer cell survival and tumor growth in vivo

**Fig. 6:**
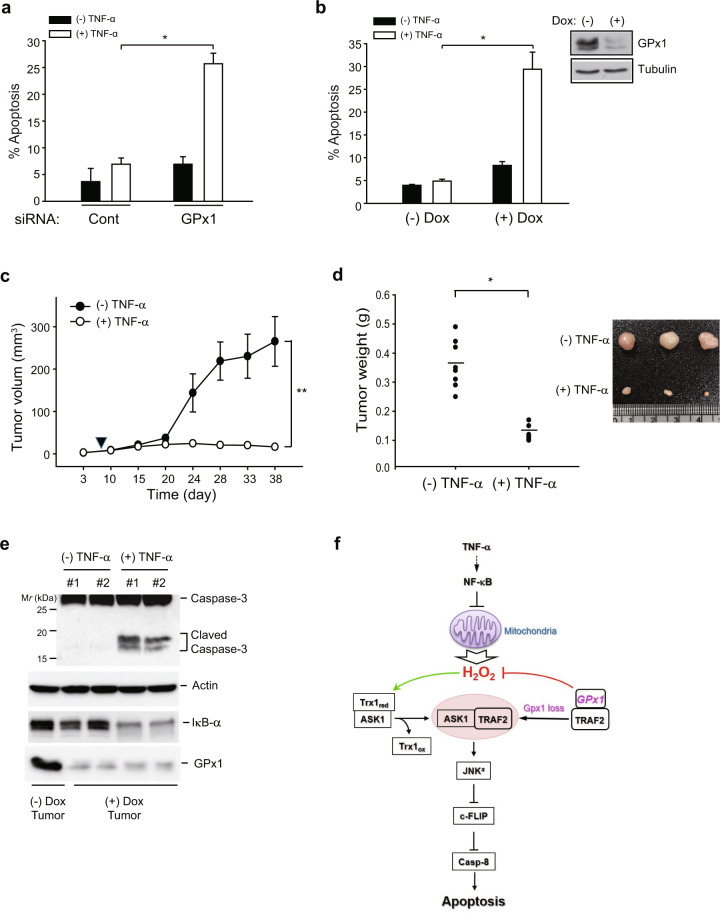
GPx1 depletion suppresses the tumorigenic growth of MDA-MB-231 cells in vivo. (**a**) The apoptosis rate was measured in siRNA-transfected HeLa cells. The transfected HeLa cells were unstimulated or stimulated with TNF-α (10 ng/ml) alone for 16 h. The data in the graph are the means ± SD of the percent of apoptotic cells (*n* = 3, ^*^P < 0.0002). **b** The apoptosis rate was measured in a Dox-inducible MDA-MB-231 cell line harboring a small hairpin GPx1 (shGPx1) sequence. MDA-MB-231 stable cells were stimulated with and without TNF-α (10 ng/ml). The data in the graph are the mean ± SD of the percent of apoptotic cells (*n* = 3, ^*^P < 0.001). A representative immunoblot shows the depletion of GPx1 upon treatment with doxycycline (Dox, 5 μg/ml) for 24 h. **c**, **d** Stable MDA-MB-231 cells were pretreated with Dox for 24 h and then subcutaneously injected into athymic *nu/nu* mice and grown for 40 days. The intravenous administration of recombinant mouse TNF-α (40 μg/kg) was started 7 days after cell injection (*filled arrowhead*) and continued twice per week for 30 days. The data in the graphs are the means ± SD of the tumor volume and weight (*n* = 8 mice per group, ^*^P < 0.005, ^**^P < 0.002 with repeated-measures ANOVA). A representative picture of tumor xenografts is shown (**d**). The systemic toxicity of TNF-α treatment was ruled out because no difference in the body weights of the mice in the two groups was observed for the duration of the experiments. **e** Caspase-3 activation was analyzed by immunoblotting the homogenates of tumor tissues obtained from two mice. GPx1 and IκB levels were immunoblotted as references. A representative immunoblot is shown. **f** Schematic model depicting the dual roles of GPx1 in ASK1 activation related to the TNF-α-induced apoptosis pathway. ASK1 is retained in the inactive form by interacting with the reduced form of thioredoxin 1 (Trx1_re_). GPx1 maintains cellular TRAF2 proteins away from ASK1 through protein-protein interaction. Upon TNF-α stimulation, the blockade of NF-κB activation triggers mitochondria-derived H_2_O_2_ production, which can be adequately controlled by cytosolic GPx1. When GPx1 expression is depleted or inhibited, the cellular H_2_O_2_ concentration is increased to a level that can oxidize thioredoxin. Then, ASK1 is freed from oxidized Trx1 (Trx1_ox_) and oligomerized upon association with the freed TRAF2. The resulting ASK1 activation induces the sustained activation of JNK (JNK^s^), which is essential for apoptosis.

Since GPx1 depletion greatly enhanced ASK1 activation, we questioned whether GPx1 depletion sufficiently sensitizes cancer cells to apoptosis upon exposure to external stress, such as cytokine storms. To address this question, we stimulated GPx1-depleted HeLa cells with TNF-α alone (without cycloheximide). Indeed, GPx1 depletion induced an increase in the apoptosis rate of HeLa cells stimulated with TNF-α alone for 16 h, but had no effect on the apoptosis rate of control cells (Fig. 6a). Since the stimulation of TNF-α alone or even TNF-α plus a pan-caspase inhibitor (zVAD) did not alter GPx1 expression, we presumed that GPx1 deficiency-induced H_2_O_2_ acts as the bona fide apoptosis trigger. Thus, we were encouraged to evaluate the in vivo function of GPx1 in a mouse xenograft model. For this in vivo study, we established a stable MDA-MB-231 cell line harboring a small hairpin RNA (shRNA) specific to GPx1, whose expression was inducible by treatment with doxycycline (Dox). Dox treatment induced the complete depletion of GPx1 expression and enhanced the TNF-α-induced apoptosis of stable MDA-MB-231 cells (Fig. 6b). Subsequently, we established subcutaneous tumor xenografts in athymic *nu*/*nu* mice using stable MDA-MB-231 cells and administered Dox to the mice twice weekly to maintain GPx1 depletion. Since a preliminary experiment showed that Dox treatment itself had no effect on the growth of MDA-MD-231-driven tumors, we analyzed tumor growth in response to TNF-α stimulation. The measurement of the tumor volume demonstrated that MDA-MB-231 tumors grew in unstimulated mice, whereas tumor growth ceased in the TNF-α-treated mice (Fig. 6c). As a result, the final tumor weight was dramatically reduced in the TNF-α-stimulated mice compared to that in the unstimulated mice (Fig. 6d). Consistently, caspase-3 activation, a hallmark of intrinsic apoptosis, was markedly induced in TNF-α-stimulated tumors compared to unstimulated tumors (Fig. 6e). Thus, the in vivo study validated the findings showing that GPx1 serves as a redox shield against apoptotic insults in tumor-residing cancer cells.

## Discussion

In mammalian cells, two major thiol peroxidase families are critical for eliminating H_2_O_2_: GPx and peroxiredoxin (Prx). The cytosolic forms of these families of enzymes seem to be functionally redundant because they exhibit similar catalytic efficiencies (~10^6^–10^7^ M^−1^s^−1^). However, in vitro enzyme kinetics studies demonstrated that GPx exhibits stable reactivity with hydroperoxides at concentrations up to one millimole^42,43^, whereas the 2-Cys Prx enzymes exhibit a submicromolar *K*_m_ for H_2_O_2_^44,45^. These differing affinities for H_2_O_2_ led us to assume a distinct role of GPx in cell death. In this study, we revealed that GPx1 regulated ASK1-dependent apoptosis via interaction with TRAF2, differing from other redox proteins involved in ASK1 activation.

The redox regulation of ASK1 activation among mitogen-activated protein kinase kinase kinase (MAP3K) family proteins is distinctive. In particular, ASK1 has been found to interact with diverse redox proteins, such as Trx1^46^, Prx1^47^, and glutaredoxin^48^. For example, Trx1-dependent regulation has been reported to be a major mode of redox-dependent ASK1 activation. ASK1 remains in a latent state by binding with Trx1 in the absence of oxidative stress. Once Trx1 is oxidized and dissociated upon oxidative stress, ASK1 is activated by TRAF2-mediated oligomerization^49^. In contrast, we demonstrate that GPx1 can suppress ASK1 activation by binding with TRAF2 upon TNF-α stimulation (Fig. 6f). This binding may induce local peroxidase activity that modulates the H_2_O_2_ concentration near the ASK1/Trx1 complex. For full activation, it has been reported that ASK1 binds to the C-terminal TRAF domain on TRAF2 and is then activated by the RING domain^40^. Similar to TRAF6, TRAF2-ASK1 activation is likely ROS-dependent, and the TRAF2 RING domain may exert E3 ligase activity^50,51^. Thus, the TRAF2 binding of GPx1 through the Zn-finger domain is a significant evolutionarily conserved regulatory mechanism because GPx1 can control the local H_2_O_2_ level and prevent Trx1 oxidation. In fact, Trx1 functions as a protein disulfide reductase that reduces diverse cytosolic and nuclear substrate proteins, including Prx, ribonucleotide reductase, and transcription factors^52^. The C^32^XXC^35^ motif essential for disulfide reduction has a p*K*a of 7.3 and thus exhibits very low reactivity to H_2_O_2_ (*K*_m_ > 5 mM)^53^. Hence, Trx1 is not considered a true peroxidase capable of competing with GPx1. However, during apoptosis, when the H_2_O_2_ level reaches an adequately high level to inactivate GPx1^3,4^, the CXXC motif in Trx1 is oxidized. In addition, the GPx1 regulation of local H_2_O_2_ level realized by binding TRAF2 is distinct from the regulatory action of Prx1, which forms a mixed disulfide with ASK1 to relay the oxidation signal^47^.

Notably, in the present study, GPx1 depletion induced the hyperactivation of JNK in HeLa and MDA-MB-231 cells. Considering that the TNF-α-induced H_2_O_2_ burst has been previously shown to cause oxidative inactivation of JNK phosphatases^54^, JNK hyperactivation is a result of the dual action of GPx1 in regulating H_2_O_2_, which leads to activated ASK1 and inactivated JNK phosphatase. Thus, GPx1 may be a multifaceted apoptosis regulator; however, the consequences of its regulatory function converge to promote sustained JNK activation.

Recently, we reported a role for GPx1 in the migratory activity of triple-negative breast cancer (TNBC) cells, including MDA-MB-231 cells^55^. In the previous study, we showed that GPx1 expression is unusually high in TNBC cells and hence regulates the proliferation and migration of TNBC cells in response to serum stimulation. In contrast, in cancer cell types with a RIPK3-negative background, the function of GPx1 switched to promoting an antiapoptotic effect. Since we observed that GPx1 deficiency enhanced TNF-α-induced apoptosis of MCF7 (luminal A type and RIPK3-negative) cells, the antiapoptotic function of GPx1 is thought to be independent of the molecular subtype of breast cancer. Overall, our studies demonstrate that the anti-apoptotic function of GPx1 is dependent on the absence of RIPK3 expression in cancer cells.

## Supplementary information

Supplementary Information
