# Supplementary material for: Glutathione peroxidase-1 regulates ASK1-dependent apoptosis via interaction with TRAF2 in RIPK3-negative cancer cells
Source: Exp Mol Med. 2021 Jun 22;53(6):1080–91. doi: 10.1038/s12276-021-00642-7 (PMC8257591; doi:10.1038/s12276-021-00642-7)
Supplement: Supplementary file 1 — Supplementary Information [file 12276_2021_642_MOESM1_ESM.pdf]

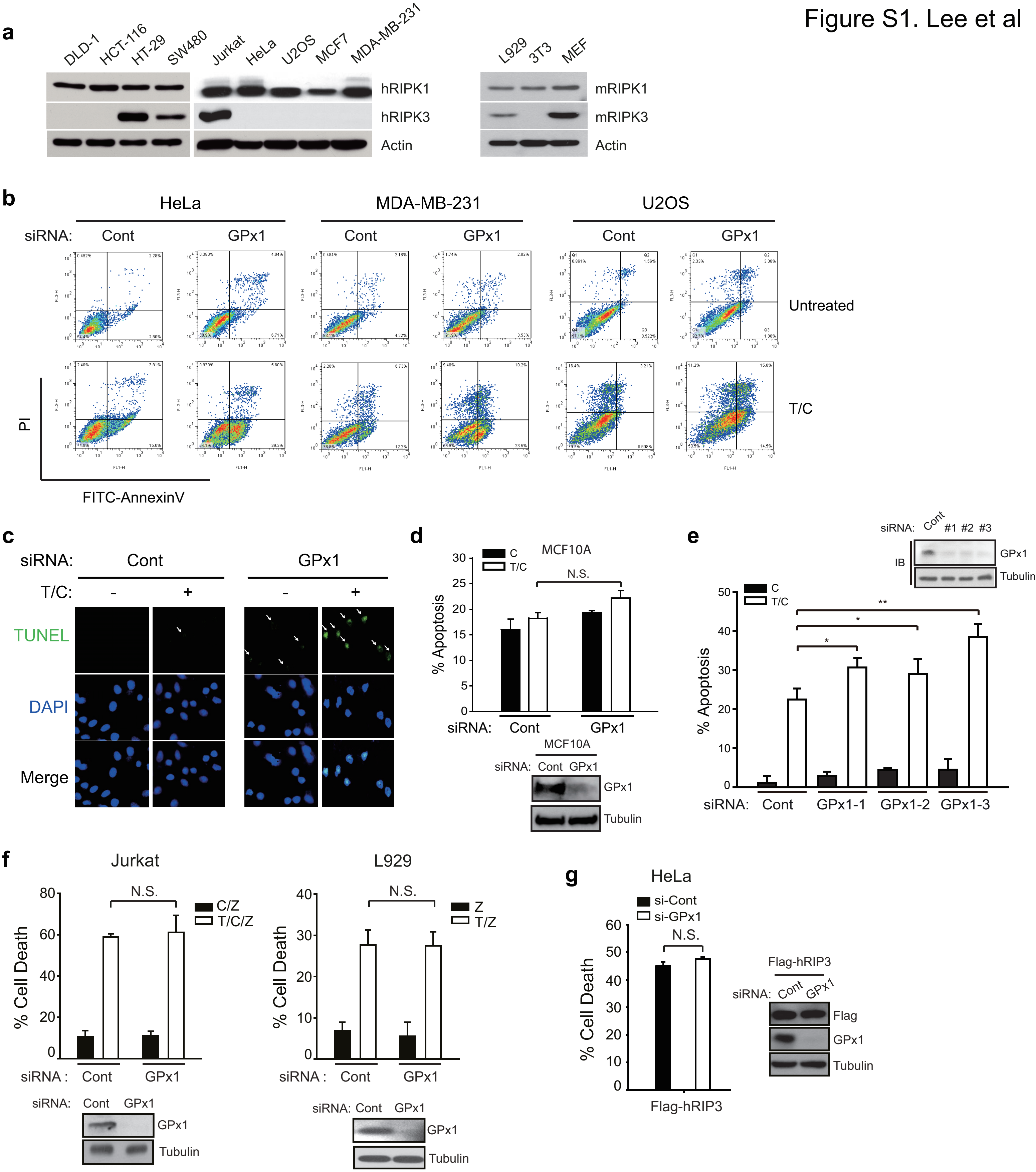

Figure S1. Effect of GPx1 on TNF-α-induced apoptosis (Related to Fig. 1).
(**a**) Expression of RIPK1 and RIPK3 protein in various cancer cell types. hRIPK and mRIPK present human and mouse RIPK protein, respectively. Actin is a loading control. (**b**) FACS analysis of the siRNA-transfected cancer cells untreated or treated with T/C for 6 h. PI, propidium iodide. (**c**) TUNEL staining of the T/C-treated HeLa cell. Arrows indicate the apoptotic cells. (**d**) Apoptotic cell death in the T/C-treated MCF10A, normal mammary epithelial cells, transfected with control or GPx1 siRNA. Note that MCF10A was not susceptible for TNF-α-induced apoptosis. (**e**) HeLa cells were transfected with three different sequences of siRNAs specific to GPx1. The knockdown level of GPx1 protein was verified by immunoblotting. The transfected HeLa cells were treated with T/C for 6 h. According to the result, the siRNA #3 is mainly used for further experiments. (**f** and **g**) TNF-α-induced necroptosis in the RIPK3-positive cancer cells. HeLa cells (**g**) were transfected with pcDNA vector encoding human RIPK3 tagged with Flag eiptope and treated with T/C/Z. ZVAD (Z) was pretreated to induced caspase-independent necroptosis in cancer cells.
Data in the graph are means ± SD of percent of apoptotic cells (n = 3, *P<0.001, **P<0.0001). Control siRNA is specific to firefly luciferase. N.S., not significant.

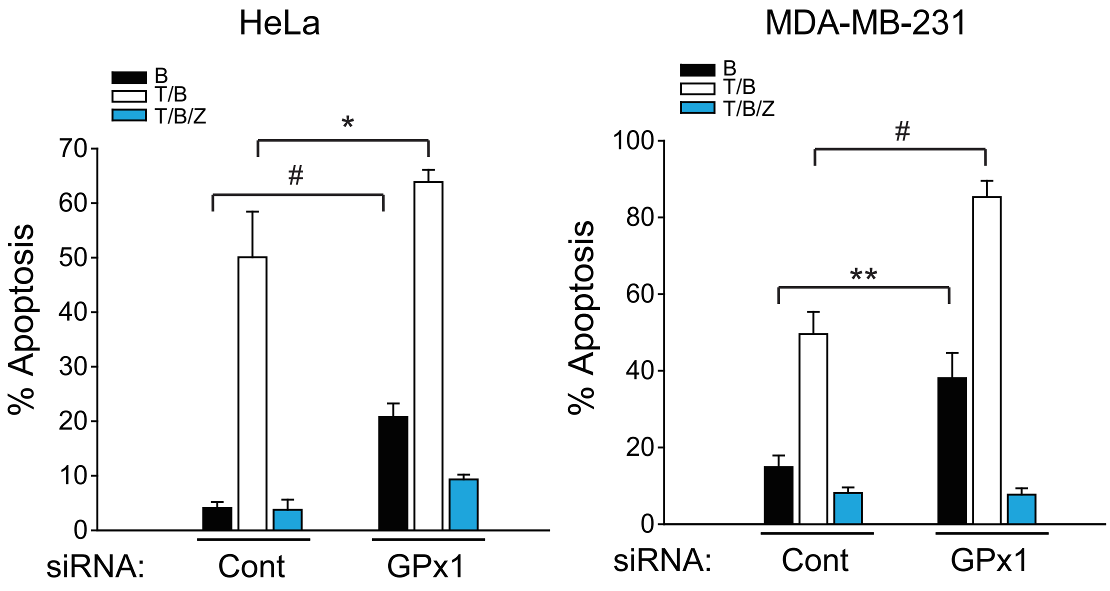

Figure S2. Effect of GPx1 on TNF-α-induced apoptosis in the Smac mimetic (Related to Fig. 1).
HeLa and MDA-MB-231 cells were transfected with control and GPx1 siRNAs and stimulated with TNF-α-in the presence of a Smac mimetic, BV6 (B) for 6 h. ZVAD (Z) was also pretreated to inhibit the apoptosis. Note that BV6 alone slightly induced apoptosis. Data in the graph are means ± SD of percent of apoptotic cells (*n* = 3, **P*<0.05, ***P*<0.005, #*P*<0.001). Control siRNA is specific to firefly luciferase.

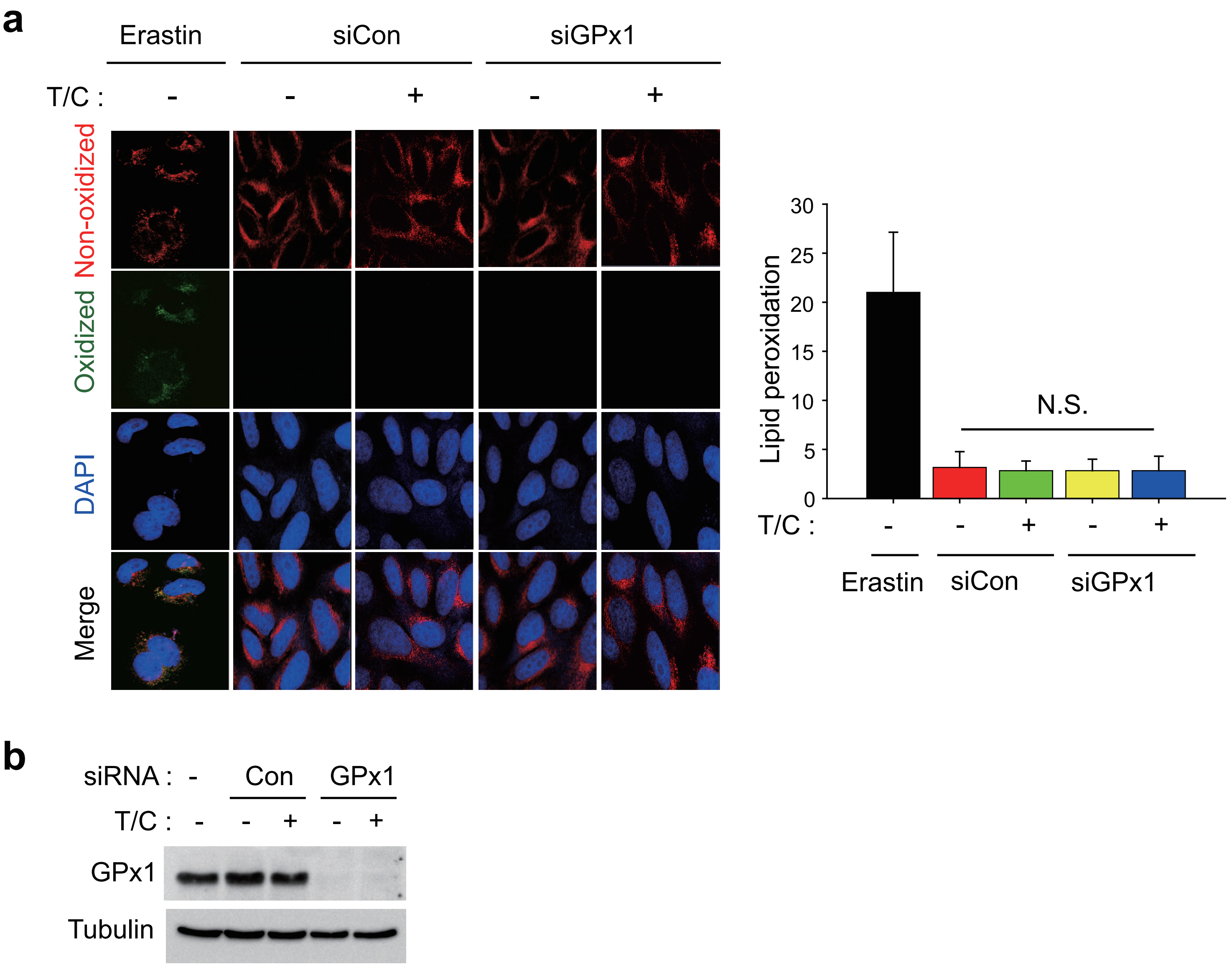

Figure S3. Level of lipid hydroperoxide in the GPx1-depleted MDA-MB-231 cells (Related to Fig. 2). The level of lipid hydroperoxide (LOOH) was measured using a LOOH-specific fluorescent probe, C11-BODIPY$^{581/591}$, in the MDA-MB-231 cells (**a**). Cells were transfected with either control or Gpx1 siRNA (siCon or siGpx1) for 48 h and treated with C11-BODIPY (1 μM). A ferroptosis inducer (Erastin) was used in parallel for control experiment. The GPx depletion was verified by immunoblotting (**b**). Data in the graph are means ± SD of the relative rations of green versus red fluorescence intensities averaged from 40 cells (*n* = 3). Representative images are shown. N.S., not significant.

Figure S4. Lee et al

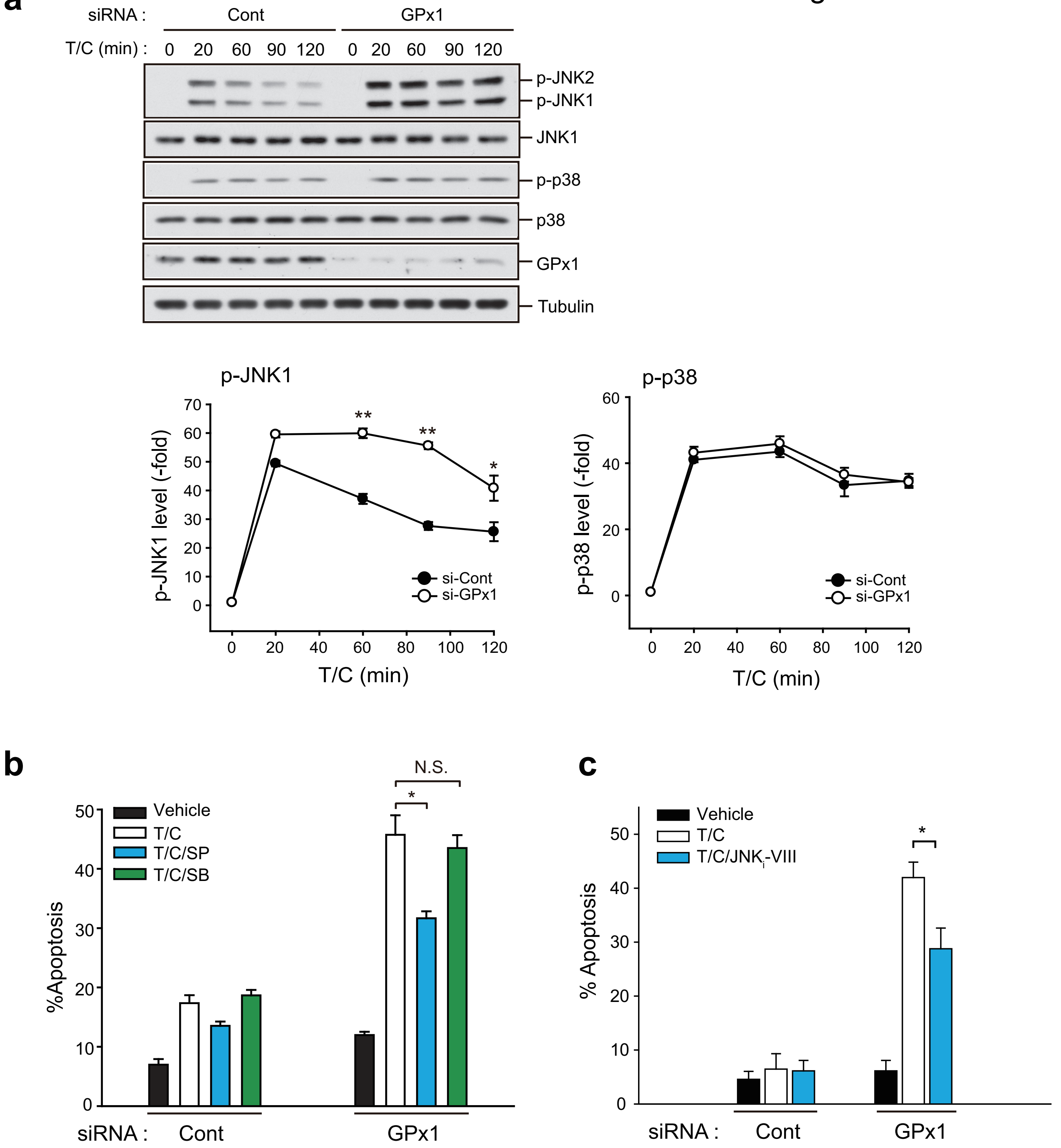

Figure S4. GPx1 differentially regulates MAPK activation in the MDA-MB-231 cells (Related to Figure 3).
(**a**) The siRNA-transfected MDA-MB-231 cells were treated with T/C and lysed for immunoblotting. The immune bands for the phosphorylated MAPKs were quantified and normalized by the intensity of corresponding MAPK bands. Data in the graph are means ± SD of fold change of band intensities (n = 3, *P<0.05, **P<0.001). An immunoblot against α-tubulin is the loading control.
(**b** and **c**) The siRNA-transfected MDA-MB-231 (**b**) and HeLa (**c**) cells were pretreated with MAPK inhibitors (10 μM each) for 30 min. Apoptosis was measured after T/C treatment for 6 h. Data in the graph are means ± SD of percent of apoptotic cells (n = 3, *P<0.01). SP600125 (SP) and JNK inhibitor VIII (JNK$_i$-VIII) are specific JNK inhibitors, while SB203880 (SB) is specific p38 inhibitor. N.S., not significant.
